# The Influence of Adult Attachment on Patient Self-Management in Primary Care - The Need for a Personalized Approach and Patient-Centred Care

**DOI:** 10.1371/journal.pone.0136723

**Published:** 2015-09-18

**Authors:** Katja Brenk-Franz, Bernhard Strauss, Fabian Tiesler, Christian Fleischhauer, Paul Ciechanowski, Nico Schneider, Jochen Gensichen

**Affiliations:** 1 Institute of General Practice and Family Medicine, University Hospital, Jena, Germany; 2 Institute of Psychosocial Medicine and Psychotherapy, University Hospital, Jena, Germany; 3 Department of Psychiatry and Behavioural Sciences, University of Washington, Seattle, United States of America; University of Western Brittany, FRANCE

## Abstract

**Objective:**

Self-management strategies are essential elements of evidence-based treatment in patients with chronic conditions in primary care. Our objective was to analyse different self-management skills and behaviours and their association to adult attachment in primary care patients with multiple chronic conditions.

**Methods:**

In the *apricare* study (Adult Attachment in Primary Care) we used a prospective longitudinal design to examine the association between adult attachment and self-management in primary care patients with multimorbidity. The attachment dimensions *avoidance* and *anxiety* were measured using the ECR-RD. Self-management skills were measured by the FERUS (motivation to change, coping, self-efficacy, hope, social support) and self-management-behaviour by the DSMQ (glucose management, dietary control, physical activity, health-care use). Clinical diagnosis and severity of disease were assessed by the patients’ GPs. Multivariate analyses (GLM) were used to assess the relationship between the dimensions of adult attachment and patient self-management.

**Results:**

219 patients in primary care with multiple chronic conditions (type II diabetes, hypertension and at least one other chronic condition) between the ages of 50 and 85 were included in the study. The attachment dimension anxiety was positively associated with motivation to change and negatively associated with coping, self-efficacy and hope, dietary control and physical activity. Avoidance was negatively associated with coping, self-efficacy, social support and health care use.

**Conclusion:**

The two attachment dimensions anxiety and avoidance are associated with different components of self-management. A personalized, attachment-based view on patients with chronic diseases could be the key to effective, individual self-management approaches in primary care.

## Introduction

Supporting self-management in patients with multiple chronic diseases is of central importance in primary care. Self-management can be defined as a behavioural and cognitive strategy that can help patients who suffer from chronic conditions to structure their behaviour, to learn problem-solving skills and how to achieve effective disease management goals [1]. The cognitive processes of self-management play an important role in therapy as they consist of components such as self-efficacy, coping and hope [2,3]. Self-efficacy for example is the central cognitive component of self-management and refers to one’s confidence in being able to perform a specific behaviour [4–6]. Evidence from clinical trials suggests that training in self-management skills are more effective than traditional patient education and can reduce treatment costs for patients with chronic conditions [6]. Furthermore, self-management is an essential element of evidence-based medical care for patients with chronic diseases in primary care [7,8]. However, to implement effective patient-centred self-management programs, it is important to understand the differences in patient interaction styles. Attachment theory provides a psychosocial model to explain individual differences in patients’ experiences and coping behaviour in relation to interpersonal closeness and distance, and affect regulation in situations that are perceived as threatening such as chronic disease [9–12]. The present study is based on a conceptual classification of the two attachment dimensions *anxiety* and *avoidance* [13]. Patients scoring high on the avoidance scale have learned to suppress their attachment needs. They tend to evade emotional closeness and intimacy and have a tendency to feel uncomfortable about opening up to or depending on others. Patients scoring high on the anxiety scale have a hyper-activated attachment system. They tend to be preoccupied with others and have a tendency to fear rejection and abandonment [14]. Previous research has proposed a model to describe the impact that insecure attachment has on the maintenance of disease or chronic conditions, e.g. through reduced self-management skills [15]. So far, there is a dearth of studies that examine the differences between the two attachment dimensions and the various components of self-management. Hence, the aim of this study was to identify the associations between adult attachment and self-management skills, and evaluate the self-management behaviour in patients with the multiple chronic diseases most common among elderly patients in surgeries of family physicians in Germany.

## Methods

### Study design and recruitment

The study was designed as a multicentre, prospective, longitudinal, observational study with patients from 8 general practices in Germany. The GPs’ electronic databases were used to identify eligible patients. Patients were recruited if they had 3 specified chronic diseases (type II diabetes, hypertension and at least another chronic condition out of a standardized list of chronic diseases [16]), were aged between 50 and 85, and consulted their GP at least once within the last 3 months. Emergency patients, patients from other family practices and those unable to give informed consent were excluded from the study. 25–35 patients in each surgery were included in the study. Patients were invited by the study physicians in their general practice to participate. Recruitment was carried out in accordance with the primary care research recruitment rules of "The German MultiCare-Study" [16]. Recruitment and baseline data collection took place between March 2012 and June 2012. The study was conducted in accordance with the "Declaration of Helsinki", the guidelines of Good Clinical Practice. It was approved by the institutional review board of the University Hospital Jena in January 2011 (No.3009-12/10). Follow-up data collection took place 12 months later, between March 2013 and June 2013.

### Data Collection

Of the eligible patients with the specified chronic diseases, 25–35 patients per practice were selected at random and invited to participate in the study by a letter from their GP. Patients were asked to contact their GP in order to give their informed consent. Participants gave written consent to participate in this study and then received the relevant documents. Both the GP and the patient signed the declaration. This consent procedure was in accordance with the ethic committee. As a result 219 patients could be included in the study. The physician completed a basic documentation and patients were given a questionnaire for self-assessment, which was completed at home and returned to the GP in a stamped addressed envelope. Patients received a remuneration of 10 euros for their efforts. The questionnaires the physicians had to fill in included the ICD-10 diagnosis and assessment of the severity of the chronic conditions. Each GP received a remuneration of 1000 euros.

### Measures

Patients’ socio-demographic data was measured based on the recommendations of the "Epidemiologic Methods" of the Association of Epidemiology [17].

#### Conditions and documentation of multimorbidity

GPs completed a list of predefined chronic diseases as that used in the German MultiCare cohort study [16]. GPs rated severity of the chronic conditions using the ‘Cumulative Illness Rating Scale for Geriatrics’ (CIRS-G) having been briefed by a physician from the research team. The CIRS-G is a multimorbidity index based on disease severity grouped at organ system levels [18]. A 4-level classification of severity is used to assess the 14 organ systems [19].

#### Adult Attachment

In the present study, adult attachment was primarily assessed using the ‘Experience of Close Relationships—Revised’ (ECR-RD) [14,20]. The ECR-RD is a dimensional measure of adult attachment style and has two subscales: avoidance and anxiety. Each subscale is rated on a seven-point Likert scale. In general, individuals with higher scores on attachment avoidance report lower intimacy. They find intimacy uncomfortable and prefer to seek independence. People scoring highly on the attachment anxiety dimension have a tendency to fear rejection and abandonment. The validation of the German version showed Cronbach's alpha reliability scores of .91 and .92 for the two relevant sub-scales [20].

#### Self-Management

The FERUS tool for the measurement of resources and self-management skills was used [3]. The FERUS includes a total of 26 items in the short version with a five-point Likert scale. The subscales are measuring motivation for change, coping, self-efficacy, self-verbalization, hope and social support. The internal consistency (Cronbach's alpha) for the subscales is between 0.86–0.93 [3]. The assessments were carried out at baseline and 12 month follow up. The ‘Diabetes Self-Management Questionnaire’ (DSMQ) was used to measure self-management behaviours (diabetes-related activities) at follow up [21]. The DSMQ assesses self-management behaviours over the past 8 weeks based on 16 items with a four-point Likert scale. The internal consistency was found to be good with Cronbach's alpha = 0.84 [21]. Self-management scores of the four subscales ‘Glucose Management’–which tests blood sugar measure, ‘Dietary Control’–how much of a diabetes-friendly diet an individual has, ‘Physical Activity’ and ‘Health-Care Use’ can be calculated [21].

### Statistical Analysis

Although the number of missing values was very low, we used procedures for the management of missing values [22]. Multiple imputation procedures are commonly seen as the most adequate method for dealing with missing values in complex data sets [23,24]. SPSS uses a Markov-Chain-Monte-Carlo algorithm known as fully conditional specification (FCS) or chained equations imputation [25]. Multiple imputation was used to generate 10 sets of ‘‘complete” data with no missing values [22]. Multivariate analysis, using the General Linear Model (GLM) was performed to analyse the association between attachment dimensions and patient self-management skills and behaviours. Multivariate analysis allows simultaneous testing of all subscales of the FERUS at baseline (t1) and at 12 month follow up (t2) and of the DSMQ at 12 month follow up (t2) as dependent variables, while considering various factors and covariates. The advantage of multivariate analyses compared with single tests is the reduction in accumulated alpha error. For overall model testing we used the Pillai's trace statistic, which is the most powerful and most robust of the four possible multivariate statistics [26]. Additional factors such as socio-demographic variables, health status, and degree of chronicity were included in the model. Moreover, regression coefficients and estimates of the effect sizes were calculated. An alpha level of p ≤ 0.05 was used for tests of statistical significance. Statistical analysis and the multiple imputations were performed using IBM SPSS 20 for Windows (Chicago, IL, USA).

## Results

A total of 219 patients (95 females) were included in the study (Table 1). The patients' ages ranged from 50 to 85, with a mean age of 66.4 years ± 8.3. The number of chronic diseases diagnosed by the GP ranged from 3 to 18, with a mean of 6.4 ± 2.5. The most common chronic diseases identified within the study population, according to ICD10 in addition to the inclusion criteria Type II Diabetes (E11) and Hypertension (ICD10 code: I10-I13) were disorders of lipoprotein metabolism (ICD10 code: E78) (52%), chronic ischaemic heart disease (ICD10 code: I20-I25) (42%) and other dorsopathies (ICD10 code: M50-M54) (36%).

**Table 1:** Characteristics of the study sample (N = 219).

Variables	Categories	Frequency		Percentage	
Age (M = 66.4; SD = 8.3)	50–61	67		30.6	
	62–73	110		50.2	
	74–85	42		19.2	
Sex	Female	95		43.4	
Marital status	Married	158		72.1	
Education	High school	152		69.4	
		Mean (SD)		Range	
Chronic Diseases	Number of chronic diseases diagnosed by the GP	6.4 (2.5)		3–18	
Multimorbidity Index (based on disease severity)	Cumulative Illness Rating Scale (CIRS) rated by the GP	8.3 (4.1)		2–38	
		**Baseline**		**Follow up**	
		**Mean**	**Range**	**Mean**	**Range**
Attachment Dimension	Anxiety	1.9 (1.3)	1–7	1.8 (1.1)	1–6
	Avoidance	2.3 (1.4)	1–6	2.2 (1.4)	1–6
Self-Management Skills (FERUS)	Motivation for Change	13.4 (5.3)	5–25	13.9 (5.7)	5–25
	Coping	18.7 (4.0)	5–25	18.8 (3.8)	8–25
	Self-Efficacy	19.2 (3.9)	9–25	19.3 (4.0)	6–25
	Self-Verbalization	14.0 (3.5)	4–20	13.9 (3.6)	4–20
	Hope	10.6 (2.9)	3–15	10.6 (2.7)	3–15
	Social Support	16.7 (3.5)	6–20	16.5 (3.4)	4–20
Self-Management Behaviour (DSMQ)	Glucose Management			7.9 (2.0)	0–10
	Dietary Control			7.3 (1.4)	3–10
	Physical Activity			7.0 (2.0)	0–10
	Health-Care Use			9.2 (1.2)	4–10
	Total DSMQ			7.8 (1.1)	4–10

Multivariate analysis with the GLM showed a significant association between the attachment dimensions of the ECR-RD (anxiety and avoidance) and both self-management skills (FERUS) and self-management behaviours (DSMQ), controlling for socio-demographic factors (age, sex and marital status), and the participants’ state of health (Number of chronic diseases diagnosed by the GP and Cumulative Illness Rating Scale/CIRS rated by the GP) (Tables 2 and 3). The attachment dimension *anxiety* showed a significant effect on baseline self-management skills (Pillai´s Trace = 0.18; F = 6.81; p≤0.001), on follow up self-management skills (Pillai´s Trace = 0.18; F = 6.50; p≤0.001) and on self-management behaviour (Pillai´s Trace = 0.11; F = 3.28; p≤0.05). Tests of between-subject effects and the parameter estimators showed a negative influence of attachment *anxiety* on the baseline subscales *coping*, *self-efficacy* and *hope*, and on the follow-up subscale *self-efficacy*. Moreover, there was a significant positive effect of the anxiety dimension on motivation for change. The attachment dimension *avoidance* showed a significant effect on baseline self-management skills (Pillai´s Trace = 0.18; F = 6.89; p≤0.001), and on follow-up self-management skills (Pillai´s Trace = 0.14; F = 4.67; p≤0.001) and self-management behaviour (Pillai´s Trace = 0.08; F = 2.32; p≤0.05). Tests of between-subject effects and the parameter estimators showed a significant negative influence of attachment *avoidance* on the baseline subscales *coping*, *self-efficacy* and *social support* and on the follow-up subscales *social support*. With regard to self-management behaviour there was a negative effect of the *anxiety* dimension on *dietary control* and *physical activity* and a negative effect of attachment *avoidance* on compliance with GP contacts (*Health-Care Use*). Regression coefficients, standard errors and p values are shown in Tables 2 and 3. No significant effect of the CIRS on self-management skills was detected. There was also no significant effect of socio-demographic characteristics and health status on self-management behaviour.

**Table 2:** Factors that influence self-management skills measured with the subscales of FERUS at baseline and follow-up; Results of the Multivariate analysis with the General Linear Model (GLM).

Predictor Variable	Motivation for change		Coping		Self-Efficacy		Self-Verbalization		Hope		Social Support	
t1	t1 B(SE)	t2 B(SE)	t1 B(SE)	t2 B(SE)	t1 B(SE)	t2 B(SE)	t1 B(SE)	t2 B(SE)	t1 B(SE)	t2 B(SE)	t1 B(SE)	t2 B(SE)
**Anxiety**	**1.54** *** **(0.27)**	**1.42** *** **(0.29)**	**-0.65** ** **(0.21)**	-0.38 (0.21)	**-0.79** *** **(0.21)**	**-0.73** *** **(0.22)**	-0.11 (0.19)	0.16 (0.21)	**-0.32** * **(0.15)**	-0.08 (0.16)	-0.19 (0.18)	-0.06 (0.19)
**Avoidance**	0.49 (0.27)	0.41 (0.29)	**-0.42** * **(0.2)**	-0.33 (0.21)	**-0.5** * **(0.2)**	-0.24 (0.22)	-0.06 (0.19)	-0,04 (0.21)	-0.26 (0.15)	-0.21 (0.16)	**-1.05** *** **(0.18)**	**-0.84** *** **(0.18)**
**Chronic Diseases**	-0.05 (0.15)	0.01 (0.17)	**-0.42** *** **(0.11)**	**-0.45** *** **(0.12)**	**-0.27** * **(0.11)**	**-0.44** *** **(0.13)**	**-0.26** * **(0.11)**	**-0.4** *** **(0.12)**	**-0.33** *** **(0.08)**	**-0.23** * **(0.09)**	**-0.24** * **(0.1)**	**-0.33** ** **(0.11)**
**CIRS**	0.05 (0.09)	0.09 (0.1)	0.00 (0.07)	-0.01 (0.07)	-0.04 (0.07)	0.02 (0.08)	0.03 (0.06)	0.05 (0.07)	-0.04 (0.05)	-0.07 (0.05)	0.06 (0.06)	0.09 (0.06)
**Age**	-0.08 (0.04)	0.02 (0.05)	0.04 (0.03)	0.04 (0.03)	0.03 (0.03)	**0.07** * **(0.03)**	**0.09** ** **(0.03)**	**0.07** * **(0.03)**	-0.03 (0.02)	-0.01 (0.02)	0.01 (0.03)	0.03 (0.03)
**Sex (dichotom)**	-0.2 (0.81)	-0.68 (0.84)	-0.02 (0.62)	-1.06 (0.62)	0.05 (0.61)	-0.48 (0.64)	0.5(0.57)	-0.21 (0.6)	0.09 (0.45)	0.14 (0.45)	0.52 (0.54)	0.36 (0.54)
**Marital status (dichotom)**	-1.33 (1.17)	-1.83 (1.28)	-0.31 (0.9)	0.67 (0.94)	0.85 (0.88)	1.15 (0.97)	-0.62 (0.82)	**-2.05** * **(0.91)**	0.54 (0.66)	0.41 (0.69)	-0.2 (0.79)	0.03 (0.82)

*p≤0.05

** P≤0.01

***p≤0.001

t1 = baseline; t2 = follow up (after 12 months)

**Table 3:** Factors that influence self-management behaviour (DSMQ) at follow-up; Results of the Multivariate analysis with the General Linear Model (GLM).

Predictor Variable	Glucose Management	Dietary Control	Physical Activity	Health-Care Use	DSMQ total
t1	t2 B(SE)	t2 B(SE)	t2 B(SE)	t2 B(SE)	t2 B(SE)
**Anxiety**	-0.01 (0.17)	**-0.26** * **(0.11)**	**-0.54** *** **(0.15)**	-0.18 (0.1)	**-0.22** ** **(0.08)**
**Avoidance**	-0.16 (0.17)	0.16 (0.11)	-0.04 (0.15)	**-0.2** * **(0.1)**	-0.08 (0.08)
**Chronic Diseases**	0.05 (0.09)	-0.01 (0.06)	-0.1 (0.08)	0.04 (0.05)	0.01 (0.04)
**CIRS**	0.00 (0.05)	0.02 (0.03)	0.07 (0.05)	-0.02 (0.03)	0.03 (0.02)
**Age**	0.01 (0.02)	0.01 (0.02)	0.02 (0.02)	0.01 (0.01)	0.01 (0.01)
**Sex (dichotom)**	-0.8 (0.44)	0.24 (0.28)	0.35 (0.4)	-0.16 (0.26)	-0.1 (0.21)
**Marital status (dichotom)**	-0.49 (0.7)	0.74 (0.45)	0.72 (0.64)	0.54 (0.41)	0.26 (0.34)

*p≤0.05

** P≤0.01

***p≤0.001

t1 = baseline; t2 = follow up (after 12 months)

## Discussion

Attachment theory provides a suitable explanatory model for individual differences in disease-related behaviour and coping [12,15,27]. Based on the attachment model’s assumptions on adult attachment and disease [15], we analysed the relationship between attachment and self-management in more detail. The results of this study suggest associations between the attachment dimensions anxiety and avoidance and self-management skills and behaviours in elderly patients with diabetes, hypertension and at least another chronic condition. The attachment dimension *anxiety* at baseline was significantly associated with higher levels of motivation for change and lower levels of coping, self-efficacy and hope also at baseline, and with higher levels of motivation for change, lower levels of self-efficacy dietary control and physical activity at 12 month follow-up.

These results may be understood in terms of the characteristics of those individuals who scored highly on the dimensions of anxious attachment. Their patterns of affect regulation and interpersonal attitudes and thus behaviour can be understood as the consequence of a hyper-activated attachment system. For people with higher levels of attachment anxiety, a chronic sense of danger triggers an amplified distress response and preoccupation with maintaining emotional and physical closeness to attachment figures, such as healthcare professionals [28]. Anxiously attached individuals tend to be dependent on others and behave in accordance with their attachment-related preoccupations. They have little trust in their own ability to cope. Self-efficacy, the belief in one’s ability to cope, plays a very important role in the self-regulation of affective states [29]. Patients with higher scores of anxious attachment tend to see themselves as ill-equipped to cope with potentially stressful events, such as the continued supported self-management of multiple chronic diseases. Thus, attachment anxiety can plausibly explain a lack of long-term illness management skills. For patients with high levels of attachment anxiety, seeking others for support is not effective in reducing their distress levels [30]. In the context of primary care, family practitioners are limited in how much support they can offer. This means that for people with higher levels of attachment anxiety, family practitioners may not be able to offer the level of support they request. On the one hand, they have social support systems and activate these intensely; on the other hand they evaluate the support negatively [27]. Attachment avoidance, however, was significantly associated with lower levels of coping, social support and self-efficacy at baseline and lower levels of social support and healthcare use at 12 month follow-up. These findings are also consistent with characteristics of people with higher levels of attachment avoidance, that is a tendency to emotionally distance themselves from others [31,32]. Attachment avoidance is characterized by the deactivation of the attachment system. Despite the experience of stress such as in the case of chronic disease management, proximity-seeking is reduced, signs of stress and help-seeking behaviour are supressed [27]. In primary care settings, mistrust and lack of self-disclosure to medical staff result in ineffective cooperation. This is characterized by trivializing symptoms, non-compliance with appointments and treatment recommendations, which are not coordinated with the GP [33,34]. Our finding of the association between avoidant attachment and health care use is consistent with previous studies [35]. There are few studies which examine the predictive role of patient attachment in the context of medical outcome variables, using a longitudinal prospective design [36,37]. We were able to confirm that attachment is a significant and useful predictor of self-management skills, such as self-efficacy and social support. Our study also shows that attachment dimensions are specific to diabetes-related behaviours, such as healthcare system use or medical outcome variables at 12 month. Thus a personalized attachment-based view beyond chronic disease can be useful for the evaluation and provision of individual self-management support in primary care.

## References

[1] König CJ, Kleinmann M (2006) Selbstmanagement In: Schuler H, editor. Lehrbuch der Personalpsychologie, 2 überarbeitete und erweiterte Auflage. Göttingen: Hogrefe.

[2] Kanfer FH, Reinecker H, Schmelzer D (2006) Selbstmanagement-Therapie: Ein Lehrbuch für die klinische Praxis (5., korrigierte und durchgesehene Auflage). Heidelberg: Springer Medizin Verlag.

[3] Jack M (2007) FERUS—Questionnaire for Measuring Resources and Self-management skills. Göttingen: Hogrefe.

[4] Bandura A (1977) Self-efficacy: toward a unifying theory of behavioral change. Psychol Rev 84: 191–215. 847061 10.1037//0033-295x.84.2.191

[5] Schwarzer R (2002) Self-efficacy In: Schwarzer R, Jerusalem M, Weber H, editors. Health Psychology from A to Z. Göttingen: Hogrefe.

[6] Bodenheimer T, Lorig K, Holman H, Grumbach K (2002) Patient self-management of chronic disease in primary care JAMA 288: 2469–2475. 12435261 10.1001/jama.288.19.2469

[7] Wagner EH, Austin BT, Davis C, Hindmarsh M, Schaefer J, Bonomi A (2001) Improving chronic illness care: translating evidence into action Health Aff (Millwood) 20: 64–78. 11816692 10.1377/hlthaff.20.6.64

[8] Gensichen J, Muth C, Butzlaff M, Rosemann T, Raspe H, de Cornejo GM, et al (2006) [The future is chronic: German primary care and the Chronic Care Model-the comprehensive principles in the proactive treatment of the chronically ill]. Z Arztl Fortbild Qualitatssich 100: 365–374. 16955622

[9] Bowlby J (1977) The making and breaking of affectional bonds. BJPsych 130: 201–210. 843768 10.1192/bjp.130.3.201

[10] Bowlby J (1988) A Secure Base Clinical Applications of Attachment Theory. London: Routledge.

[11] Berman WH, Marcus L, Berman ER (1994) Attachment in marital relations In: Sperling MB, Berman WH, editors. Attachment in adults: theory, assessment and treatment. New York: Guilford Press.

[12] Schmidt S, Nachtigall C, Wuethrich-Martone O, Strauß B (2002) Attachment and coping with chronic disease. J Psychosom Res 53: 763–773. 12217450 10.1016/s0022-3999(02)00335-5

[13] Bartholomew K, Horowitz LM (1991) Attachment styles among young adults: a test of a four-category model. J Pers Soc Psychol 61: 226–244. 1920064 10.1037//0022-3514.61.2.226

[14] Fraley RC, Waller NG, Brennan KA (2000) An item response theory analysis of self-report measures of adult attachment. J Pers Soc Psychol 78: 350–365. 10707340 10.1037//0022-3514.78.2.350

[15] Maunder RG, Hunter JJ (2001) Attachment and psychosomatic medicine: developmental contributions to stress and disease. Psychosom Med 63: 556–567. 11485109 10.1097/00006842-200107000-00006

[16] Schafer I, Hansen H, Schon G, Maier W, Hofels S, et al (2009) The German MultiCare-study: Patterns of multimorbidity in primary health care—protocol of a prospective cohort study. BMC Health Serv Res 9: 145 10.1186/1472-6963-9-145 19671164 PMC3224741

[17] Jöckel KH, Babitsch B, Bellach BM, Bloomfield K, Hoffmeyer-Zlontnik JHP, Winkler J (1998) [Recommendations of the working group "Epidemiologic Methods" in the German Association Epidemiology of GMDS and DGSMP to measure and quantify the socio-demographic characteristics in epidemiological studies]. In: Ahrens W, Bellach BM, Jöckel KH, editors. [Measurement of socio-demographic characteristics in epidemiology]. Munich: MMV-Verlag pp. 7–38.

[18] Miller MD, Paradis CF, Houck PR, Mazumdar S, Stack JA, Rifai AH (1992) Rating chronic medical illness burden in geropsychiatric practice and research: application of the Cumulative Illness Rating Scale. Psychiatry Res 41: 237–248. 1594710 10.1016/0165-1781(92)90005-n

[19] Hudon C, Fortin M, Soubhi H (2007) Abbreviated guidelines for scoring the Cumulative Illness Rating Scale (CIRS) in family practice. J Clin Epidemiol 60: 212 17208130 10.1016/j.jclinepi.2005.12.021

[20] Ehrenthal JC, Dinger U, Lamla A, Funken B, Schauenburg H (2009) Evaluation der deutschsprachigen Version des Bindungsfragebogens "Experiences in Close Relationships—Revised" (ECR-RD). Psychother Psychosom Med Psychol 59: 215–223. 10.1055/s-2008-1067425 18600614

[21] Schmitt A, Gahr A, Hermanns N, Kulzer B, Huber J, Haak T (2013) The Diabetes Self-Management Questionnaire (DSMQ): development and evaluation of an instrument to assess diabetes self-care activities associated with glycaemic control. Health Qual Life Outcomes 11: 138 10.1186/1477-7525-11-138 23937988 PMC3751743

[22] Schafer JL (1997) Analysis of incomplete multivariate data. Chapman & Hall: London.

[23] Rubin DB (1987) Multiple Imputation for Nonresponse in Surveys. New York: J. Wiley & Sons.

[24] Rubin DB (1996) Multiple imputation after 18+ years (with discussion). JASA: 473–489.

[25] Gilks WR, Richardson S, Spiegelhalter DJ (1996) Markov Chain Monte Carlo in Practice. London: Chapman and Hall.

[26] Olson CL (1976) On choosing a test statistic in multivariate analysis of variance. Psychological Bulletin 83: 579–586.

[27] Mikulincer M, Shaver PR (2007) Attachment in adulthood: structure, dynamics, and change. New York: Guilford.

[28] Mikulincer M, Shaver PR, Pereg D (2003) Attachment theory and affect regulation: The dynamics, development, and cognitive consequences of attachment-related strategies. Motiv Emot 27: 77–102.

[29] Bandura A (1997) Self-efficacy: the exercise of control New York: Freeman.

[30] Campbell L, Simpson JA, Boldry J, Kashy DA (2005) Perceptions of conflict and support in romantic relationships: the role of attachment anxiety. J Pers Soc Psychol 88: 510–531. 15740443 10.1037/0022-3514.88.3.510

[31] Mikulincer M, Nachshon O (1991) Attachment styles and patterns of selfdisclosure. J Pers Soc Psychol 61: 321–331.

[32] Rholes WS, Simpson JA, Stevens JG (1998) Attachment orientations, social support, and conflict resolution in close relationships In: Simpson JA, Rholes WS, editors. Attachment theory and close relationships. New York: Guilford Press pp. 166–188.

[33] Ciechanowski PS, Walker EA, Katon WJ, Russo JE (2002) Attachment theory: a model for health care utilization and somatization. Psychosom Med 64: 660–667. 12140356 10.1097/01.psy.0000021948.90613.76

[34] Ciechanowski P, Katon W, Hirsch I (1999) Attachment style and adherence in the diabetic patient. Psychosom Med 61: 110.

[35] Ciechanowski PS, Hirsch IB, Katon WJ (2002) Interpersonal predictors of HbA(1c) in patients with type 1 diabetes. Diabetes Care 25: 731–736. 11919133 10.2337/diacare.25.4.731

[36] Tuck NL, Consedine NS (2013) Adult attachment and emotion regulation: better predictors of subjective or objective health? J Behav Health 2: 308–317.

[37] Henderson SJ (2009) Attachment Security as a Predictor of Blood Glucose Control in Adolescents with Type 1 Diabetes, when the Roles of Additional Psychological Factors are Considered. Edinburgh: University of Edinburgh.

